# System-level performance measures of access to rheumatology care: a population-based retrospective study of trends over time and the impact of regional rheumatologist supply in Ontario, Canada, 2002–2019

**DOI:** 10.1186/s41927-022-00315-6

**Published:** 2022-12-27

**Authors:** Claire E. H. Barber, Diane Lacaille, Ruth Croxford, Cheryl Barnabe, Deborah A. Marshall, Michal Abrahamowicz, Hui Xie, J. Antonio Avina-Zubieta, John M. Esdaile, Glen Hazlewood, Peter Faris, Steven Katz, Paul MacMullan, Dianne Mosher, Jessica Widdifield

**Affiliations:** 1 Department of Medicine, University of Calgary, Calgary, AB Canada; 2 Department of Community Health Sciences, University of Calgary, AB Calgary, Canada; 3 McCaig Institute for Bone and Joint Health, University of Calgary, Calgary, AB Canada; 4 Arthritis Research Canada, Vancouver, BC Canada; 5 Department of Medicine, University of British Columbia, Vancouver, BC Canada; 6 ICES, Toronto, Canada; 7 Department of Epidemiology and Biostatistics, McGill University, Montreal, QC Canada; 8 Faculty of Health Sciences, Simon Fraser University, Burnaby, BC Canada; 9 Alberta Health Services, Calgary, AB Canada; 10 Department of Medicine, University of Alberta, Edmonton, AB Canada; 11 Institute of Health Policy, Management and Evaluation, University of Toronto, Toronto, Canada; 12 Holland Bone and Joint Program, Sunnybrook Research Institute, Toronto, Canada

**Keywords:** Rheumatoid arthritis, Quality care, Access to care, Performance measure

## Abstract

**Objective:**

To determine whether there were improvements in rheumatology care for rheumatoid arthritis (RA) between 2002 and 2019 in Ontario, Canada, and to evaluate the impact of rheumatologist regional supply on access.

**Methods:**

We conducted a population-based retrospective study of all individuals diagnosed with RA between January 1, 2002 and December 31, 2019. Performance measures evaluated were: (i) percentage of RA patients seen by a rheumatologist within one year of diagnosis; and (ii) percentage of individuals with RA aged 66 years and older (whose prescription drugs are publicly funded) dispensed a disease modifying anti-rheumatic drug (DMARD) within 30 days after initial rheumatologist visit. Logistic regression was used to assess whether performance improved over time and whether the improvements differed by rheumatology supply, dichotomized as < 1 rheumatologist per 75,000 adults versus ≥1 per 75,000.

**Results:**

Among 112,494 incident RA patients, 84% saw a rheumatologist within one year: The percentage increased over time (adjusted odds ratio (OR) 2019 vs. 2002 = 1.43, *p* < 0.0001) and was consistently higher in regions with higher rheumatologist supply (OR = 1.73, 95% CI 1.67–1.80). Among seniors who were seen by a rheumatologist within 1 year of their diagnosis the likelihood of timely DMARD treatment was lower among individuals residing in regions with higher rheumatologist supply (OR = 0.90 95% CI 0.83–0.97). These trends persisted after adjusting for other covariates.

**Conclusion:**

While access to rheumatologists and treatment improved over time, shortcomings remain, particularly for DMARD use. Patients residing in regions with higher rheumatology supply were more likely to access care but less likely to receive timely treatment.

**Supplementary Information:**

The online version contains supplementary material available at 10.1186/s41927-022-00315-6.

## Background

Timely diagnosis and treatment for rheumatoid arthritis (RA) is critical for optimizing patient outcomes [1, 2]. National RA system-level performance measures (PMs) have been developed to measure access to rheumatologists and treatment in Canada [3]. Previous evaluations of the PMs using provincial health administrative data in Alberta [4], British Columbia [5] and Ontario [6] have demonstrated gaps in care including poor access to rheumatologist care and suboptimal dispensation of appropriate therapy with disease modifying anti-rheumatic drugs (DMARDs). Furthermore, there remain concerns about the supply of rheumatologists nationally, regionally [7] and internationally [8, 9], and it is unclear to what degree this impacts access to RA care.

Ontario is Canada’s largest province with over 14 million inhabitants [10]. In the last few decades, the number of rheumatologists in the province has increased, although the growth in rheumatology supply has not kept pace with population growth. Further, rheumatology workforce demographic changes (increasing feminization and workforce aging [11]) may impact access to care. Ontario is also geographically large (> 1 million km^2^) and there is regional variation in numbers of rheumatologists with clustering around southern urban centers [12], which may affect access to care for individuals living outside urban centers. Our aim was to determine whether there were improvements in access to rheumatology care and timeliness of DMARD treatment between 2002 and 2019 in the province, and to evaluate the impact of rheumatologist regional supply on these performance measures.

## Patients & methods

### Study design and setting

We conducted a population-based inception RA cohort study in Ontario. Ontario residents receive all medically necessary health services free at the point of care under a single payer healthcare system. The Ontario Drug Benefit Plan covers the cost of prescription medications for Ontarians aged 65 years and older, subject to a small copayment. Health services are recorded in administrative databases which enable comprehensive evaluations of care.

### Data sources

The Ontario Health Insurance Plan (OHIP) Claims History Database was used to identify physician services and associated diagnoses. Encoded physician identifiers in the claims were linked to the ICES Physician Database to identify physician specialty and practice location. Patient demographic information, place of residence and vital status were ascertained from the OHIP Registered Persons Database. Pharmacy claims from the Ontario Drug Benefit Program were available for individuals $\ge$66 years (allowing up to one year for coverage registration). Additional information on patient health care utilization and comorbidities were obtained from hospital discharge records in the Discharge Abstract Database and emergency department visits recorded in the National Ambulatory Care Recording System database. A complete list of databases can be found in Additional file 1: Table S1. These datasets were linked using unique encoded identifiers and analyzed at ICES (www.ices.on.ca). ICES is a prescribed entity under Sect. 45 of Ontario’s Personal Health Information Protection Act. The use of data in this study was approved by a privacy impact assessment at ICES and authorized under Sect. 45 of Ontario’s Personal Health Information Protection Act, which does not require review by a Research Ethics Board.

### Patient selection

All incident RA patients between January 1, 2002 and December 31, 2019 were identified from the Ontario Rheumatoid Arthritis Database [13]. Patients are included if they have at least one hospitalization, or at least two physician claims for RA over 2 years (ICD9 714.0 or ICD10 M05-M06) with at least one from a rheumatologist, internist, or orthopedic surgeon. The case definition has been validated to have a sensitivity of 78%, specificity of 100% and positive predictive value of 78% [14].

Individuals were excluded if they had missing demographic information, a diagnosis date before 18 years of age, were non-Ontario residents at the date of their first RA code (i.e. individuals from a another province and received care in Ontario given reciprocal agreements for care coverage between provinces), or did not have health insurance eligibility in the 5-year period prior to their first RA code (to ensure only incident RA cases were included). Individuals who did not have at least 1 year of follow-up after their first RA code were also excluded. (Flow diagram of cohort selection shown in Additional file 1: Fig. S1).

### Cohort characteristics

The following characteristics were determined at cohort entry: age, sex, neighborhood income quintiles derived from census data, and urban versus rural location of residence (the latter defined based on postal code and a community size of < 10,000 residents) [15] Fourteen Local Health Integration Networks (LHINs), as defined by the Ontario Ministry of Health, were used as geographic health service regions. Linear distance in kilometers between patients and the nearest rheumatologist was determined (calculated from the center of the patient’s postal code), and individuals residing 100 km from the nearest rheumatologist were described as living at a “remote” distance. Comorbidities in the three years prior to RA diagnosis were assessed using diagnosis codes from physician claims and hospital discharge records and applying validated health administrative algorithms [16–21] when available. In addition, the Johns Hopkins ACG^®^ System version 11 was used to assign each patient to up to 32 Aggregated Diagnosis Groups^®^ (ADGs) using diagnosis codes found in OHIP physician claims and hospital discharge records, using a three-year look-back period. A patient frailty indicator was also obtained using the ACG System.

### System performance measure (PM) adherence

Two PMs [3] were operationalized as previously described [4, 5], and assessed between 2002 and 2020 for patients entering the cohort in each year. The first PM reports on access to rheumatologist care and is calculated as the percentage of incident RA patients seen by a rheumatologist within 365 days of their first RA diagnosis code by any physician.

The second PM is reported on individuals aged 66 and older who saw a rheumatologist within the first year of diagnosis (measured from the first RA code) and lived at least an additional 30 days. This PM reports on the percentage of individuals dispensed a DMARD following diagnosis confirmation at the rheumatologist visit [3, 22]. Given potential clinical challenges in starting treatment within the recommended benchmark of 14-days [3, 22] (e.g., due to delays in obtaining baseline lab investigations and/or receiving appropriate medication counselling, time for appropriate patient decision making, or patient delays in filling a prescription), we applied a 30-day benchmark. DMARDs in these analyses included conventional synthetic DMARDs, targeted synthetic DMARDs, biologic agents as well as other immunosuppressive therapies used to treat complications of RA (see Additional file 1: Table S2 for included DMARDs).

### Regional rheumatology supply

Based on recommendations from the Canadian Rheumatology Association [3], regional rheumatologist supply was classified as optimal (at least one rheumatologist per 75,000 residents in the region of the patients’ residence) or suboptimal (less than one rheumatologist per 75,000) based on the local health region (LHIN).

### Statistical analysis

We used descriptive statistics (mean or median depending on normality of the data, and frequencies) to characterize patients at cohort entry. Outcomes were assessed annually as the proportion of incident patients diagnosed each year who met each of the PMs, stratified by rheumatologist supply. Logistic regression was used to assess whether there were improvements in trends over time, and whether the improvements were associated with rheumatologist supply. Multivariable models additionally adjusted for factors which may affect access to care, including age at diagnosis, sex, income quintile, rural residence, log(distance to nearest rheumatologist), and comorbidities. Time from diagnosis to first rheumatologist visit was included in the model predicting DMARD use, and a generalized estimating equation was used to account for the clustering of patients within rheumatologist practices. Because the trends over time were not linear, the shape of the trend was characterized using fractional polynomials [23]. In the adjusted analyses, fractional polynomials were also used to characterize the relationship between patient age and both PMs. All analyses were performed using SAS version 9.3 (SAS Institute Inc., Cary, NC). Two-tailed* p*-values < 0.05 were considered to be statistically significant.

## Results

Demographic characteristics for the entire cohort of 112,494 individuals and for the subset of individuals aged 66 and older are shown in Table 1. Most (n = 94,812 (84.3%)) saw a rheumatologist within 1 year of their first RA diagnosis code. The percentage was significantly (*p* < 0.0001) smaller for individuals aged 66 and older (‘seniors’); only 30,020 (77.4%) of 37,823 seniors saw a rheumatologist within one year of their first RA diagnosis code versus 64,792 (86.7%) of 74,671 non-seniors. (Table 1)

**Table 1:** Characteristics of incident RA patients

Variable	All ages	Age 66 and older	Age 66 and older, subset who saw a rheumatologist within 1 year of diagnosis
	N = 112,494	N = 37,823	N = 30,020
Number (%) of Females	77,134 (68.6%)	24,648 (65.2%)	19,453 (64.8%)
Mean age at diagnosis (SD)	58.1 (15.5)	74.8 (6.4)	74.5 (6.2)
*Neighborhood income quintiles (1 = low, 5 = high)*			
1	21,028 (18.8%)	6924 (18.4%)	5286 (17.7%)
2	22,798 (20.3%)	7809 (20.7%)	6122 (20.5%)
3	23,052 (20.6%)	7797 (20.7%)	6189 (20.7%)
4	22,824 (20.4%)	7471 (19.8%)	6004 (20.1%)
5	22,364 (20.0%)	7710 (20.4%)	6334 (21.2%)
Number (%) living in rural areas	15,307 (13.6%)	5196 (13.8%)	3955 (13.2%)
Remote (> 100 + km) to nearest rheumatologist	5665 (5.0%)	1776 (4.7%)	913 (3.0%)
Mean (SD) rheumatologists per 75,000 adult population	1.66 ± 1.11	1.70 ± 1.14	1.76 ± 1.14
Median (Q1, Q3) distance (km) to nearest rheumatologist (km)	5 (2, 23)	5 (2, 25)	4 (2, 19)
Patients living in region with at least one rheumatologist per 75,000 adults	82,619 (73.4%)	28,242 (74.7%)	23,427 (78.0%)
*Comorbidity*			
AMI	1209 (1.1%)	736 (2.0%)	515 (1.7%)
Coronary artery disease	7830 (7.0%)	5304 (14.0%)	4022 (13.4%)
Cancer	10,479 (9.3%)	6197 (16.4%)	4883 (16.3%)
Cardiovascular disease	3483 (3.1%)	2386 (6.3%)	1700 (5.7%)
CHF	1489 (1.3%)	1167 (3.1%)	776 (2.6%)
Chronic renal disease	2552 (2.3%)	1736 (4.6%)	1325 (4.4%)
COPD or asthma	10,076 (9.0%)	4599 (12.2%)	3571 (11.9%)
Diabetes	15,689 (14.0%)	8128 (21.5%)	6340 (21.1%)
DVT or pulmonary embolism	567 (0.5%)	305 (08%)	228 (0.8%)
Hypertension	33,413 (29.7%)	18,925 (50.0%)	14,830 (49.4%)
*Number of ADGs*			
< 5	10,428 (9.3%)	2216 (5.9%)	1712 (5.7%)
5–9	45,336 (40.3%)	12,972 (34.3%)	10,316 (34.4%)
10–14	43,358 (38.5%)	16,187 (42.8%)	12,927 (43.1%)
15+	13,372 (11.9%)	6448 (17.1%)	5065 (16.9%)
Frailty*	5,557 (4.9%)	3829 (10.1%)	2657 (8.9%)
Outcomes			
Saw rheumatologist within 1 year of diagnosis	94,812 (84.3%)	30,020 (77.4%)	30,020 (100%)
*Time from diagnosis to first visit to a rheumatologist, for those who saw a rheumatologist within 1 year*			
0 days	59,694 (63.0%)		19,253 (64.1%)
1–183 days	31,340 (33.1%)		9,590 (32.0%)
184–365 days	3778 (4.0%)		1177 (3.9%)
Filled a prescription for a DMARD within 30 days of first visit to a rheumatologist			18,863 (62.8%)

*ADG* Aggregated diagnosis group (Johns Hopkins ACG^®^ System);* AMI* Acute myocardial infarction;* CHF* Congestive heart failure;* COPD*  Chronic obstructive pulmonary disease;* DMARD*  Disease modifying antirheumatic drug;* DVT*  Deep vein thrombosis

*An individual with a diagnosis related to malnutrition, dementia, impaired vision, decubitus ulcer, incontinence of urine or feces, loss of weight, obesity, poverty, barriers to access of care, or difficulty walking is classified as frail. Urquart R, Giguere AMC, Lawson B, Kendell C, Holroyd-Leduc JM, Puyat JH et al. Rules to identify persons with frailty in administrative health databases. Can J Aging. 2017; 36(4): 514–521.

**Fig. 1:**
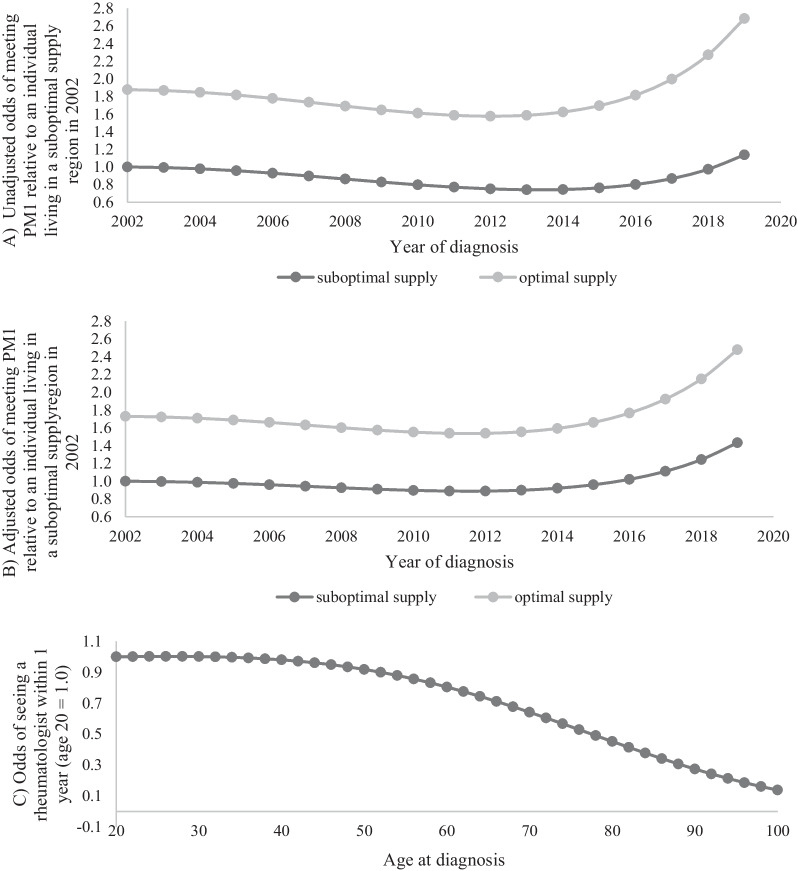
Trends over time and age for visit to a rheumatologist within one year of diagnosis (PM1).** A** Unadjusted trends over time, by regional rheumatologist supply (suboptimal supply: $<$ 1 rheumatologist / 75,000 adults; high supply: ≥ 1 rheumatologist/75,000 adults).** B** Adjusted trends over time, by regional rheumatologist supply. ** C** Adjusted access to a rheumatologist, by age. Models were adjusted for age at diagnosis, sex, income quintile, rural residence, log(distance to nearest rheumatologist) and comorbidities.

The unadjusted regression found a net improvement in the percentage of patients who saw a rheumatologist within one year of diagnosis over the study period, despite an initial downward trend. There was a significant interaction between year and rheumatologist supply (*p* < 0.0001) such that individuals living in optimal supply regions were consistently more likely to see a rheumatologist in the first year, with the difference widening over time (Fig. 1A). Overall, between 2002 and 2019, the percentage of individuals seeing a rheumatologist within one year of diagnosis increased from 68.8 to 81.5% in regions with suboptimal rheumatologist supply (net change = 12.7%, 95% confidence interval 9.3−16.1%) while in optimal supply regions the percentage increased from 85.1 to 89.4% (net change = 4.3%, 95% confidence interval 2.8−5.7%). In 2019, the difference between regions of optimal supply and regions of suboptimal supply was 7.9% (95% confidence interval 5.6−10.2%). This pattern persisted after adjusting for patient characteristics. The interaction between year and rheumatologist supply was no longer significant after adjusting for patient characteristics (*p* = 0.54), but the net improvement and the advantage of living in region with optimal rheumatology supply persisted (Fig. 1B; Table 2). Those diagnosed at a younger age, males, people living in higher-income neighborhoods and those living in rural areas were more likely to meet the PM; those living further from the nearest rheumatologist, frail individuals and individuals with congestive heart failure, diabetes or hypertension were less likely to meet the PM (Table 2). The effect of patient age was non-linear: a decrease in the odds of meeting the PM began at around age 35 (Fig. 1C).

**Table 2:** Results of multivariable model of the probability of seeing a rheumatologist within 1 year of diagnosis

Predictor	Odds ratio	95% confidence interval	*p*-value
Year	See Fig. 2B		< 0.0001
Resident in optimal rheumatologist supply region (reference is suboptimal supply)	1.73	1.67–1.80	< 0.0001
Age	See Fig. 2C		< 0.0001
Male (reference is females)	1.11	1.07–1.15	< 0.0001
Income quintile (per additional quintile)	1.11	1.09–1.12	< 0.0001
Rural residence (reference is urban)	1.77	1.68–1.87	< 0.0001
Log(distance to nearest rheumatologist, km)	0.73	0.72–0.74	< 0.0001
Congestive heart failure	0.66	0.59–0.75	< 0.0001
Diabetes	0.91	0.87–0.95	< 0.0001
Hypertension	0.91	0.88–0.95	< 0.0001
Frailty	0.67	0.63–0.72	< 0.0001

Acute myocardial infarction (AMI), coronary artery disease cancer, cardiovascular disease, chronic renal disease, chronic obstructive pulmonary disease (COPD) or asthma, deep vein thrombosis (DVT) or pulmonary embolism, and ADG category were not significant predictors in this model. The interaction between year and rheumatologist supply was not significant (p = 0.54)

**Fig. 2:**
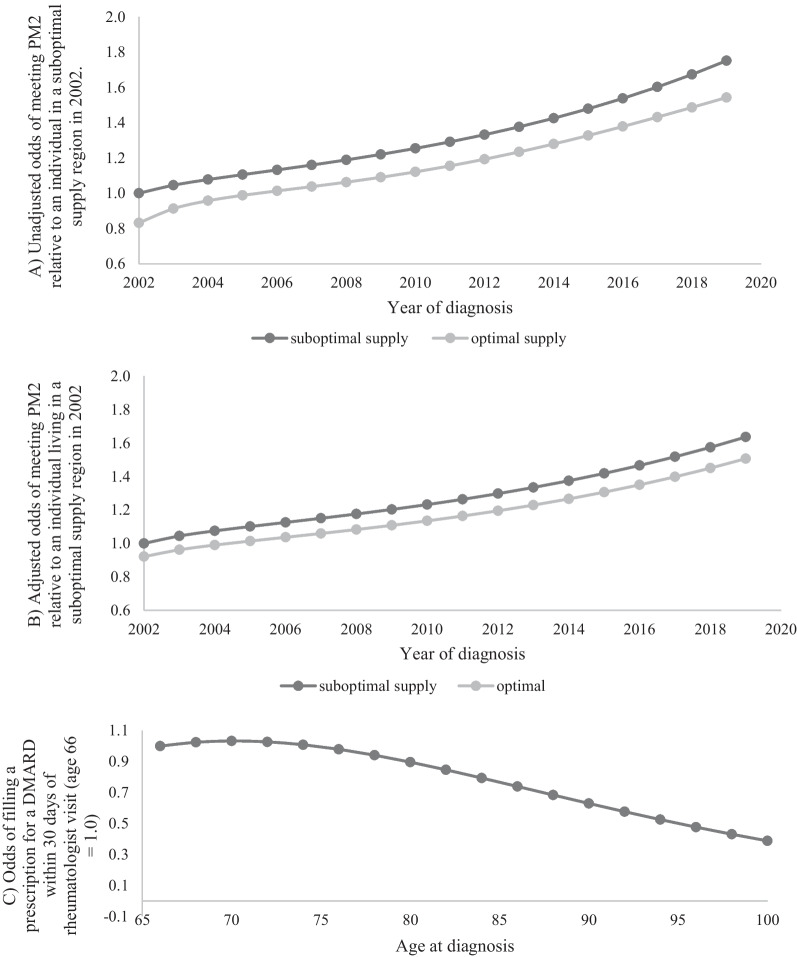
Trends over time and age for prescription for a DMARD within 30 days of first visit to a rheumatologist, for seniors (aged 66 and older at the time of diagnosis) who saw a rheumatologist within one year of diagnosis (PM2). **A** Unadjusted trends over time, by regional rheumatologist supply (suboptimal supply = < 1 rheumatologist/75,000 adults; optimal supply = ≥ 1 rheumatologist /75,000 adults). **B** Adjusted trends over time, by regional rheumatologist supply. **C** Adjusted access to a rheumatologist, by age. Models were adjusted for age at diagnosis, sex, income quintile, rural residence, log (distance to nearest rheumatologist) and comorbidities.

For the second PM (DMARD dispensation within 30 days of rheumatologist visit), the analysis was limited to seniors who had publicly funded drug coverage. Overall, DMARD dispensations were suboptimal; 62.8% of seniors who saw a rheumatologist within one year of diagnosis also received a timely prescription for a DMARD (Table 1), but performance improved consistently over the study period (Fig. 2A). Individuals living in regions with less than 1 rheumatologist per 75,000 adults were more likely to receive a DMARD prescription within 30 days of their initial rheumatology consultation (odds ratio 1.28 vs. individuals living in regions with at least one rheumatologist per 75,000 adults, 95% confidence interval 1.21−1.36, *p* < 0.0001). Between 2002 and 2019, the percentage of seniors who received a timely DMARD prescription increased from 55.2 to 68.7% (change = 13.5%, 95% confidence interval 10.0−17.0%). Across all years, 66.5% of seniors in low supply regions but 61.8% in high supply regions received a DMARD prescription on or within 30 days of rheumatology consult (difference = − 4.5%, 95% confidence interval − 5.9% to − 3.3%). These results persisted after adjusting for patient characteristics (Table 3; Fig. 2B). Additionally, the odds of meeting this PM decreased with increasing patient age (Fig. 2C), increasing time to initial rheumatologist visit, and increasing comorbidity (Table 3). However, in this group of seniors who had seen a rheumatologist within one year of their RA diagnosis, there was no indication that sex (*p* = 0.80), neighborhood income quintile (*p* = 0.13) or rurality (other than that related to number of rheumatologists) (*p* = 0.95) were related to this PM.

**Table 3:** Results of multivariable model evaluating whether a prescription for a DMARD was filled within 30 days after first rheumatology visit, among seniors who saw a rheumatologist within one year of their diagnosis

Predictor	Odds ratio	95% confidence interval	*p*-value
Year	Fig. 2B		< 0.0001
Resident in an optimal supply region (reference is low supply)	0.90	0.83–0.97	0.0059
Time from diagnosis to first rheumatologist visit			< 0.0001
0 days (reference)			
1–183 days	0.66	0.61–0.72	< 0.0001
184–365 days	0.50	0.43–0.58	< 0.0001
Age	Figure 2 C		< 0.0001
CHF	0.94	0.73–0.97	0.014
Chronic renal disease	0.80	0.71–0.89	< 0.0001
Frailty	0.91	0.84–0.99	0.026
Number of ADGs			0.0042
<5 (reference category)			
5–9	0.93	0.85–1.02	0.14
10–14	0.88	0.80–0.97	0.0083
≥ 15	0.83	0.74–0.93	0.0016

*CHF* Congestive heart failure;* DMARD *Disease modifying anti-rheumatic drug;* ADGs* Aggregated diagnosis group

The model was adjusted for clustering by physician

The following covariates were not significant in this model: sex, income quintile, rural place of residence, distance to nearest rheumatologist, chronic diseases: acute myocardial infarction, coronary artery disease, cancer cardiovascular disease, COPD or asthma, diabetes, deep vein thrombosis or pulmonary embolism, hypertension. The interaction between supply and year is also not significant in the multivariable model (*p* = 0.23)

## Discussion

High quality care for RA patients requires access to rheumatologist care for confirmation of diagnosis and prompt institution of appropriate therapy. Our prior work in Ontario has demonstrated a fairly stable number of rheumatologists (0.8 full-time equivalents (FTE)/75,000 population) [24]. However, there are regional variations in the workforce, [12] and the impact on care was unknown. While there is a trend in this contemporary era for improvement in access to rheumatologists, and in seniors, an improvement over time in timeliness of DMARD dispensation. Despite this, gaps in care remain including ongoing suboptimal DMARD dispensations to seniors with RA who have seen a rheumatologist. Additionally, over 10% of RA patients are not seen by a rheumatologist within one year of diagnosis. This is most likely a significant underestimate (and the delay may exceed one year) given we did not have referral data and relied on the use of RA specific diagnosis codes. It is likely primary care physicians use less specific diagnosis codes prior to rheumatologist confirmation of diagnosis as most patients were diagnosed as RA at the time of their initial rheumatologist visit. The benchmark in Canada for timely rheumatologist evaluation for RA is four weeks [3]; however, without accessible referral data, physician billing data was used as a proxy measure for access indicating where major health system challenges exist in rheumatologist access and potential determinants of these challenges. This work also demonstrates that social determinates of health including income quintile, rural residence, distance to the rheumatologist and sex all impacted access to rheumatology care; however, these factors did not appear to impact DMARD dispensation in seniors indicating more equitable care once individuals had rheumatology access.

The Canadian consensus-based benchmark for rheumatologist supply is one rheumatologist per 75,000 adults [3, 25]. While ideally this benchmark is applied to FTE rheumatologists, there remain challenges in identifying appropriate benchmarks for FTE that adequately estimate workforce capacity, and it is possible our results may have differed for some regions if FTEs were used. Nevertheless, when performance on the measures was stratified by rheumatologist regional supply using simple counts, we documented differences in access to care. This suggests a natural target for improvements in rheumatology human resource allocation. The Canadian Rheumatology Association (CRA) is currently developing a position statement to develop solutions to address national and regional rheumatologist deficits. The CRA endorses various workforce strategies increasing recruitment of rheumatologists, improving regional distribution, enhancing retention, promoting capacity development through recruitment of interdisciplinary health providers, supporting further research into the rheumatology workforce including developing definitions for FTE for future research and quality monitoring purposes, and enhancing equity, diversity, and inclusion in the workforce.

In seniors with RA, older age is associated with lower receipt of appropriate and timely DMARD treatment. This is compounded by the impact of additional age-related comorbid conditions and/or frailty, possibly as a result of contraindications to therapy but perhaps also as a result of characteristics of disease presentation [26]. We also observed decreased likelihood of meeting this performance measure with an increasing time to rheumatologist consultation. We hypothesize that this finding may be driven by individuals with less active disease who are evaluated with less urgency. Interestingly, the likelihood of being dispensed a DMARD within 30 days was lower in regions with optimal rheumatology supply (at least 1 rheumatologist per 75,000 population) compared to regions with suboptimal supply. There are many potential explanations for this finding, including the possibility that rheumatologists in lower supply areas may be more likely to prescribe DMARDs right away for individuals due to known access challenges, and those in higher supply regions may hold off on prescriptions as patients are more likely to be able to return for a further follow-up to discuss treatment plans. Alternatively, those travelling longer distances to see a rheumatologist may have worse symptoms and require expedited treatment (likely due to additional delays in diagnosis occurring prior to seeing a rheumatologist in areas with low rheumatology supply). In other work leveraging linkage to primary care data [27], regional variations within the province did not correlate well with rheumatologist supply as patients were often evaluated by rheumatologists outside of their local health regions. Lastly, there may be residual confounding or other bias to explain our findings. For example, collider bias [28] could be present whereby being elderly may be associated with living in an area with a larger number of rheumatologists and also being prescribed fewer DMARDs due to comorbidities Alternatively, it is possible that individuals residing in areas of high rheumatologist supply experience other challenges accessing DMARD therapy due to determinants of health not captured in this study [29].

While our work informs the relationship between measure performance and rheumatologist supply, there remain several limitations. Firstly, there are inherent limitations to administrative data including the potential for misclassification despite a validated case definition for the cohort including at least one billing code by a rheumatologist, internist, or orthopedic surgeon ( PPV > 78%) and for the second PM all individuals had seen a rheumatologist (as this PM measures the interval between the initial rheumatology visit, and DMARD dispensation). Our population-based sample was confined to all individuals with a RA diagnosis code confirmed by a musculoskeletal specialist in order to minimize misclassification bias. RA patients who remain undiagnosed, never seek health care and/or receive specialist referrals for their RA will not be represented in this analysis. Clearly the inclusion of these individuals in the population would lower the performance on the reported measures in the entire population as we have shown in prior provincial evaluations [4, 5]. Disease activity is not captured in administrative data and may impact the urgency of rheumatology referral, treatment timeliness and could impact performance measure results. Medication data was also not available on the population aged under 65 years, therefore our analysis of time to DMARD initiation was limited to those aged 66 years and older. Our findings on this PM may therefore not be generalizable to younger ages were higher DMARD use is anticipated.

## Conclusion

In conclusion, our study reveals improvements over time in access to rheumatologists for RA diagnosis and early treatment in Ontario. However, there remain gaps in access to timely diagnosis and treatment, particularly for patients living in regions with lower rheumatologist supply who were found to have reduced access to timely rheumatology care. These findings underscore the importance of enhancing resource allocation, and models of care delivery to ensue equity in access to care across regions. Further work, possibly including primary data collection, is needed to better identify system and patient-level factors impacting time to DMARD treatment.

## Supplementary Information

**Additional file 1: Table S1.** List of administrative databases used in this study. **Fig. S1.** Cohort Creation Flow-chart. **Table S2.** Complete list of disease modifying anti-rheumatic drugs (DMARDs) paid for by the Ontario Drug Benefit Plan

## Data Availability

the data are housed at ICES and are available through ICES upon appropriate request and approvals (https://www.ices.on.ca/DAS/Public-Sector/Access-to-ICES-Data-Process). Please contact author C. Barber or J. Widdifield if access required.

## Abbreviations

ADG: Aggregated Diagnosis Group
CI: Confidence interval
CRA: Canadian Rheumatology Association
DMARD: Disease modifying anti-rheumatic drug
ICES: https://www.ices.on.ca/
ICD: International classification of diseases
LHIN: Local Health Integration Network
PM: Performance measure
OHIP: Ontario Health Insurance Plan
OR: Odds ratio
RA: Rheumatoid arthritis
