# Supplementary material for: System-level performance measures of access to rheumatology care: a population-based retrospective study of trends over time and the impact of regional rheumatologist supply in Ontario, Canada, 2002–2019
Source: BMC Rheumatol. 2022 Dec 27;6:86. doi: 10.1186/s41927-022-00315-6 (PMC9793576; doi:10.1186/s41927-022-00315-6)
Supplement: Supplementary file 1 — Additional file 1: Table S1. List of administrative databases used in this study. Fig. S1. Cohort Creation Flow-chart. Table S2. Complete list of disease modifying anti-rheumatic drugs (DMARDs) paid for by the Ontario Drug Benefit Plan [file 41927_2022_315_MOESM1_ESM.docx]

**Supplemental Material**

**Supplemental Table 1** List of administrative databases used in this study

The following administrative databases were used to identify the RA cohort, and to obtain baseline patient characteristics and the two performance measure (PM) outcomes (visit to a rheumatologist within 1 year of RA diagnosis and receipt of a prescription for a DMARD within 30 days of first visit to a rheumatologist)

| Database name | Brief description of contents | Use |
| --- | --- | --- |
| Discharge Abstract Database | Contains a record of every acute care hospital discharge. Records include dates of admission and discharge, and diagnoses associated with the hospitalization. | Cohort identification  Baseline characteristics |
| ICES Physician Database | Contains information (specialty, location of practice) for physicians providing care to Ontario residents. Using an encoded physician number, information in the IPDB can be linked to OHIP claims (below) to determine the specialty of the physician providing care to a patient. Postal code of physician’s practice was used to calculate distance from each patient to the nearest rheumatologist (see Postal Code Conversion File, below). | Identification of rheumatologist visits for the performance measure (outcome)  Baseline characteristics |
| National Ambulatory Care Reporting System | Contains a record of every visit to an emergency department. Records include date of visit and diagnoses associated with the visit. | Baseline characteristics |
| Ontario Health Insurance Plan claims database | Contains records of services provided by physicians and allied professionals, including date, diagnosis, and encoded physician identification | Cohort identification  Identification of visits to a rheumatologist (outcome) |
| Ontario Drug Benefits database | Contains a record of each prescription covered by the Ontario Drug Benefits plan. All seniors (aged 65 and older) are covered by the plan. Each record identifies the drug dispensed (see Drug Information Database above) and the date. | Identification of DMARD use for the performance measure (outcome) |
| Postal Code Conversion File | Maps postal codes to information on neighborhood characteristics and location. | Baseline characteristics |
| Registered Persons Database | Has basic demographic information (date of birth, sex) on each person covered by the Ontario Health Insurance Program. The database records patient postal code information, which is linked to the Postal Code Conversion File (above) to assign neighborhood income quintile, rurality, and distance to care. | Baseline characteristics |

**Supplemental Figure 1** Cohort Creation Flow-chart

Removed for exclusion criteria: age <18, missing at least 5 years of OHIP eligibility, death on or before diagnosis date. N=11,761

Individuals in the ORAD database diagnosed between January 1, 2002, and December 31, 2019. N=126,835

N=115,074

**N=112,494 used in the analysis of access to a rheumatologist**

Individuals aged 66 and older on date of RA diagnosis N=37,823

Exclude those individuals who do not have at least 1 yearof follow-up after RA diagnosis N=2,580

For analysis of those individuals 66 and older for whom medication data is available, exclude those younger than age 66 on their diagnosis date N=74,671

Exclude those individuals who did not see a rheumatologist within 1 year of their diagnosis date N = 7,803

All who remain lived for ≥30 days after their first rheumatologist visit

**Cohort of individuals 66 and older with a full year of follow-up who saw a rheumatologist within 1 year N=30,020 used in the analysis of DMARD prescriptions**

| **Supplemental Table 2**- Complete list of disease modifying anti-rheumatic drugs (DMARDs) paid for by the Ontario Drug Benefit Plan | |
| --- | --- |
| **DMARDs and other immunosuppressive agents** | Azathioprine  Chloroquine  Cyclophosphamide  Cyclosporine  Gold  Hydroxychloroquine  Leflunomide  Methotrexate  Minocycline  Mycophenolate mofetil  Sulfasalazine  Penicillamine |
| **Biologic agents** | Abatacept  Adalimumab  Anakinra  Certolizumab  Etanercept  Golimumab  Infliximab  Rituximab  Tocilizumab |
| **Oral small molecule inhibitor** | Tofacitinib  Baricitinib  Upadacitinib |
